# Exposure assessment in investigations of waterborne illness: a quantitative estimate of measurement error

**DOI:** 10.1186/1742-5573-3-6

**Published:** 2006-05-26

**Authors:** Andria Q Jones, Catherine E Dewey, Kathryn Doré, Shannon E Majowicz, Scott A McEwen, David Waltner-Toews

**Affiliations:** 1 Division of Community Health and Humanities, Faculty of Medicine, Memorial University of Newfoundland, The Health Sciences Centre, St. John's, Newfoundland, A1B 3V6, Canada; 2 Department of Population Medicine, University of Guelph, 50 Stone Road West, Guelph, Ontario, N1G 2W1, Canada; 3 Foodborne, Waterborne and Zoonotic Infections Division, Public Health Agency of Canada (160 Research Lane, Suite 206 Guelph, Ontario, N1G 5B2, Canada

## Abstract

**Background:**

Exposure assessment is typically the greatest weakness of epidemiologic studies of disinfection by-products (DBPs) in drinking water, which largely stems from the difficulty in obtaining accurate data on individual-level water consumption patterns and activity. Thus, surrogate measures for such waterborne exposures are commonly used. Little attention however, has been directed towards formal validation of these measures.

**Methods:**

We conducted a study in the City of Hamilton, Ontario (Canada) in 2001–2002, to assess the accuracy of two surrogate measures of home water source: (a) urban/rural status as assigned using residential postal codes, and (b) mapping of residential postal codes to municipal water systems within a Geographic Information System (GIS). We then assessed the accuracy of a commonly-used surrogate measure of an individual's actual drinking water source, namely, their home water source.

**Results:**

The surrogates for home water source provided good classification of residents served by municipal water systems (approximately 98% predictive value), but did not perform well in classifying those served by private water systems (average: 63.5% predictive value). More importantly, we found that home water source was a poor surrogate measure of the individuals' actual drinking water source(s), being associated with high misclassification errors.

**Conclusion:**

This study demonstrated substantial misclassification errors associated with a surrogate measure commonly used in studies of drinking water disinfection byproducts. Further, the limited accuracy of two surrogate measures of an individual's home water source heeds caution in their use in exposure classification methodology. While these surrogates are inexpensive and convenient, they should not be substituted for direct collection of accurate data pertaining to the subjects' waterborne disease exposure. In instances where such surrogates must be used, estimation of the misclassification and its subsequent effects are recommended for the interpretation and communication of results. Our results also lend support for further investigation into the quantification of the exposure misclassification associated with these surrogate measures, which would provide useful estimates for consideration in interpretation of waterborne disease studies.

## Background

Exposure assessment is typically the greatest weakness of epidemiologic studies of disinfection by-products (DBPs) in drinking water [1-4]. This largely stems from the difficulty in obtaining accurate data on individual-level water consumption patterns and activity. In these investigations, an individual's residential address is often used to classify their home water source; this in turn, is used as a surrogate for their actual drinking water source. For instance, in several investigations of adverse birth outcomes, the maternal residential address was linked to a water system and the water quality data for that system was used to classify the individuals' exposure to DBPs [5-11]. Similarly, several studies of cancer outcomes assigned drinking water exposure by linking the case to a water system using the residential address at diagnosis or death [12-14].

Without collecting individual-level water consumption data however, there is an inherent assumption with this method of exposure classification that the home water source correctly represents the individual's actual drinking water source. Inaccuracy of such surrogate measures of waterborne exposure however, is likely to arise from several sources, including individual variability in daily tap water consumption, water consumed at work/outside of the home, residential mobility and the use of bottled water or treatment devices [1-4]. A few investigations of waterborne chemical contaminants did collect some individual-level data on water consumption, but did not collect data on other factors that can affect exposure, for instance, the use of water treatment devices [10,15-17]. Two recent studies improved the accuracy of exposure assessment of DBPs by collecting individual-level data on water consumption outside of the home and the use of bottled water and water treatment devices [18,19].

Inexpensive and convenient surrogate measures for home water source and drinking water source are useful when direct information is not available. However, without estimating the potential exposure misclassification associated with these surrogates, we cannot know the accuracy of a study's results. Assessment of these surrogate measures is especially important given the costly public policy implications of recommending changes to drinking water supplies based on health outcomes, or the costs associated with not detecting waterborne hazards.

In this study, we examine the use of two surrogate measures for home water source, namely: (a) urban/rural status as assigned using residential postal codes, and (b) mapping of residential postal codes to municipal water systems within a Geographic Information System (GIS). These surrogates were chosen because they use easily obtainable data, involve straightforward methodology and because residential addresses/postal codes are commonly used to classify waterborne exposure in studies of waterborne disease [6-8,11,15,16,21-27]. Further, we examined the use of a surrogate commonly used in the DBP literature [6-8,10,11,13-17,22,23,27], namely, an individual's home water source as a surrogate measure of their actual drinking water source.

Our study population was the City of Hamilton, Ontario (Canada), which has a diverse population of approximately 500,000. The majority of residences are served by one of five municipal water systems; however a small proportion (approximately 20%) is served by private water supplies, including private wells and water cisterns.

## Methods

### Study design and questionnaire

A cross-sectional study investigating enteric illness among residents of the City of Hamilton, Ontario (Canada), was performed between February 2001 and February 2002 [28]. A subsection of this study began in September 2001 that explored the drinking water consumption patterns in the community [29]. The study methodology is described elsewhere [28]. Briefly, a telephone questionnaire was administered, in English, to a random sample of residents of the City of Hamilton, between September 2001 and March 2002. The sampling frame was a commercial database of residential telephone numbers of households in Hamilton (SelectPhone, InfoUSA, Inc.). One individual within each household was randomly chosen to participate in the survey by selecting the individual whose birthday fell next in time. Among the data collected, respondents reported the amount of water consumed in total, and in the home, as well as the amount of commercially bottled water consumed in total, and in the home, over the previous 24-hour period. Water consumption was defined to include plain water as well as that used in the preparation of cold beverages. Total daily water intake, in this study, refers only to water consumed as such, and excludes that used in preparation of hot beverages and food. Respondents also reported whether their household used any in-home water treatment devices to treat their tap water, including jug filters, tap filters, heat, light or ion-based devices. We also asked about the source of water for the home, specifically, whether it was a private well, municipal water, both, other (specified) or unknown (to the respondent).

### Surrogates for home water source

To assess the accuracy of the two surrogate measures of home water source (urban/rural status as assigned using residential postal codes and mapping of residential postal codes to municipal water systems using a GIS), urban and rural residencies were used as surrogates for municipal and private water sources, respectively. Municipal water refers to that supplied by the area's municipal government, whereas private water refers to that from privately owned and operated wells and water cisterns.

The urban/rural status of the residents was assigned post-interview using their reported residential postal code and a Postal Code Conversion File [30]. Statistics Canada (2003) defines urban areas as those with a minimum population of 1,000 and a population density of at least 400/km^2^; all other areas are considered rural. For the second surrogate, we linked respondents to municipal or private water sources using their reported residential postal codes and digitized maps of municipal water treatment system distribution areas. Specifically, the City of Hamilton's water treatment utilities provided maps to detail their distribution areas, which were re-digitized and imported into ArcView GIS (Environmental Systems Research Institute, Inc.) polygons. If the residence's postal codes fell within municipal polygons, they were coded as having a municipal source. They were otherwise coded as private. For both surrogates, we calculated the sensitivity, specificity and predictive values to assess its performance, using self-reported home water source as the gold standard. Analyses were performed using only those residences served by municipal or private systems exclusively.

### Home water source as a surrogate for drinking water source

To examine the accuracy of using an individual's home water source as a surrogate measure of his or her actual drinking water source, we first calculated the proportion of the respondent's total daily water intake that was consumed (a) at home, from all sources (i.e. regular tap, home-treated tap and commercially bottled water), and (b) specifically from the home water source (i.e. regular tap and home-treated tap only). The latter was our first assessment of using home water source as a surrogate for an individuals' actual drinking water source. Secondly, we estimated the proportion of total daily water intake that was consumed as unmodified water from the home water source (i.e. regular tap water only). Specifically, we cross-tabulated categorized proportions of total daily water intake that were from the home water source with the use of in-home water treatment devices. "Unmodified" water, by definition, excludes water treated within the home; hence, only households not using treatment devices were included in the estimation of the proportion of total water intake consumed as unmodified water. This proportion represents the consumption of water from the home water source in its strictest sense, and was our second assessment of the surrogate.

All statistical analyses were performed in STATA for Windows version 7.0 [31]. The Human Subjects Committee at the University of Guelph and the Research Ethics Board of St. Joseph's Hospital and McMaster University approved the study.

## Results

The overall response rate for the study was 37.4% (1757/4703); however, unavailable data for some variable combinations resulted in certain analyses being performed with smaller samples, as noted below. The self-reported residential postal codes were unknown for 158 respondents, and were invalid or not recognized in the Postal Code Conversion File for another 71. Approximately 91.6% (1575/1719) and 7.9% (136/1719) of respondents reported receiving their household water from a municipal water system and private water system, respectively. Approximately 0.1% (2/1719) of respondents received their household water from a combination of municipal and private water systems and 0.4% (6/1719) reported "other" sources; these respondents were excluded from further analyses.

### Surrogates for home water source

The agreement between the two surrogate measures and the self-reported home water source are summarized in Table 1. For both surrogates, there were proportionally more rural respondents and private system-coded respondents on municipal systems than there were urban respondents and municipal system-coded respondents on private systems. Overall, the surrogate measures performed with high sensitivity when classifying residents on municipal water systems (Table 1). They did not perform as well however, in classifying individuals on private water systems (Table 1).

**Table 1:** Performance of two surrogate measures for home water source (a) residential postal code-based urban/rural status and (b) GIS-assigned home water source, using resident-reported home water source as the gold standard

**Home Water Source**	**Self-reported Municipal Water Source**	**Self-reported Private Water Source**	**Total**
**Postal-Code Based Urban/Rural Status**			
Urban designation	1318	21	1339
Rural designation	48	95	143
Total	1366	116	1482
			
Sensitivity	0.96		
Specificity	0.82		
Positive Predictive Value^†^	0.98		
Negative Predictive Value*	0.66		
			
**GIS-mapping to Water System**			
Municipal system assignment	1310	27	1337
Private system assignment	56	88	144
Total	1366	115	1481
			
Sensitivity	0.96		
Specificity	0.77		
Positive Predictive Value^§^	0.98		
Negative Predictive Value^Φ^	0.61		

^†^The proportion of urban-designated residences that truly had municipal water systems

* The proportion of rural-designated residences that truly had private water systems

^§ ^The proportion of residences assigned to municipal water systems that truly had municipal water systems

^Φ^The proportion of residences assigned to private water systems that truly had private water systems

### Home water source as a surrogate for drinking water source

Data for both in-home water treatment device use and the proportion of total daily water intake that was from the home water source were available for 1597 respondents. The cross-tabulation of these two variables is summarized in Table 2. The categorized proportions of respondents' total daily water intake that was (a) from the home water source, in general (i.e. regular tap and home-treated tap), and (b) unmodified water from the home water source (i.e. regular tap only) are summarized in Table 3. Approximately half (48%; 770/1597) of the respondents consumed all of their drinking water, and 62% (991/1597) consumed 50% or more of their drinking water, from their home water source (i.e. as regular tap and/or home-treated tap water). Approximately one-third (506/1597) of respondents did not consume any water (in any form) from their home water source. Further, unmodified water from the home water source (i.e. regular tap water) represented 100%, and 50–100%, of the total water intake for approximately 21% (343/1597) and 28% (443/1597) of respondents, respectively. Approximately 69% (1104/1598) of respondents did not use regular tap water from their home water source as their drinking water source.

**Table 2:** Cross-tabulation of the proportion of total daily water intake that was from the home water source by the use of in-home water treatment devices

**Proportion of total daily water intake that was from home water source (%)**	**In-home water treatment device used**		**Total**
		
	**Yes**	**No**	
100	427	343	770
75 to 99	45	25	70
50 to 74	76	75	151
25 to 49	41	41	82
Less than 25	8	10	18
0	186	320	506
Total	783	814	1597

**Table 3:** Proportions of total daily water intake that were from the home water source

	**Count (%)**	**Cumulative %**
**Proportion of total daily water intake that was from the home water source (%)**		
100	770 (48.2)	48.2
75 to 99	70 (4.4)	52.6
50 to 74	151 (9.5)	62.1
25 to 49	82 (5.1)	67.2
Less than 25	18 (1.1)	68.3
0	506 (31.7)	100
Total	1597	
Proportion of total daily water intake that was consumed as regular tap water from the home water source* (%)		
100	343 (21.5)	21.5
75 to 99	25 (1.6)	23.1
50 to 74	75 (4.7)	27.8
25 to 49	41 (2.6)	30.4
Less than 25	10 (0.6)	31.0
0	1103 (69.0)	100
Total	1597	

* Water from the home water source that was not further treated or modified through the use of in-home water treatment devices

## Discussion

This study examined the use of two surrogate measures for home water source, as well as the use of home water source as a surrogate for the actual drinking water source. This validation study was conducted in one North American community, over a period of six months; hence, there are likely limits to the extent to which the results may be generalized. Nevertheless, this study may serve as a rough estimate of the potential error associated with using surrogates for waterborne exposure and demonstrates that study-specific validation studies could make a difference in the study conclusions.

We considered self-reported home water source to be the best choice for the gold standard, however some misclassification may have occurred if people were unsure as to whether they paid for their household water (e.g. residents who rent) or whether they used a private water source. However, 98% of the respondents provided a response for this question despite being given the opportunity to indicate they did not know; we therefore have reason to think misclassification of our gold standard to be unlikely.

Overall, the accuracy of the two postal-code based surrogates for home water source was poor. While they provided good classification of residents served by municipal systems, they did not perform as well in classifying those served by private water systems; hence, their use in waterborne exposure classification methodology should be done with caution. The relatively poor performance of the GIS mapping surrogate in classifying private water systems was surprising and may relate to the nature of the maps and the re-digitization process. Further studies assessing the use of these surrogates are needed before conclusions regarding their accuracy can be made.

Despite its common use in the waterborne disease literature, we found that individuals' home water sources were not good surrogates for their actual drinking water source(s) in this study population. If individual-level data were not used in an investigation, and the home water source was used as the individuals' actual drinking water source, just half of the respondents would be perfectly classified. The accuracy of the surrogate measure increases as the assumed proportion of the total daily water intake that is water from the home water source decreases; for instance, half of the water intake came from the home water source for about 60% of the subjects. However, the potential for misclassification error remains significant as roughly one-third of respondents did not consume any water from the home water source. Although our study was limited to one community, these results raise serious concerns about the use of home water source as a surrogate for the actual water being consumed.

To illustrate the potential implications of such misclassification, we prepared a simple example in which we applied our exposure misclassification estimates to the data reported in one study in the literature. Gallagher et al. conducted a case-control study comparing waterborne trihalomethane exposure in a series of adverse of birth outcomes and a referent group of normal deliveries; we chose to use the results from the study that produced the highest odds ratios (Table 4) [7]. Exposure was based on maternal residence and analyses of finished municipal water samples for trihalomethanes. Our estimate suggests that, for every one truly exposed individual the exposure classification method will on average, misidentify as exposed approximately one other non-exposed individual. We applied this estimated error to the reported data and calculated the measure of association; these are the true values that would generate the observed data for the given sample size, if the actual number of false positives were as close as possible to the expected value. These estimates were then compared to the estimate of effect reported by Gallagher et al. (Table 4). In making this comparison, we assumed that the misclassified persons should have been in the lowest exposure category, which presumably would be consistent with the misclassification arising from bottled water and water treatment device use. For all exposure categories, the odds ratio tended toward the null (Table 4). We recognize that 0.5, rounded from the estimate of 0.48 in our calculations, may not be the best estimate for this population and the assumption of no false negatives is likely erroneous; again, we use this example to illustrate the potential implications of our finding.

**Table 4:** Association of trihalomethane exposure and term low birth weight (a) as reported by Gallagher et al. [7] and (b) after adjusting for 50% exposure misclassification

**Exposure Category (ppb)**	**Number of outcomes**	**Number of normal deliveries**	**Crude OR**
**(a) Original Study**			
≤ 20*	11	614	Reference
21–40	8	346	1.3
41–60	4	188	1.2
≥ 61	6	67	5.0
All exposed	18	601	1.7
**(b) With Adjustment for 50% Exposure Misclassification**			
≤ 20*	20	914	Reference
21–40	4	173	1.1
41–60	2	94	1.0
≥ 61	3	34	4.0
All exposed	9	301	1.4

Furthermore, depending on the disease being investigated, some home treatment devices might change the level of the suspect hazard in the water. For instance, ion-exchange units and boiling can change the concentrations and types of chemical contaminants in drinking water [32-34], and the misuse of some devices can increase the chemical and/or microbial contamination of the water [35,36]. Depending on the hazard being investigated, one may therefore need to take into account the use of in-home water treatment devices. The misclassification associated with using home water source as a surrogate for water consumed unmodified from the home water source was very high. For example, in this population, a study conducted on the basis that the individuals' entire daily water intake was regular tap water from the home water source would be associated with 78% misclassification. A study that assumed it represented 50% or more of individuals' drinking water intake would correctly classify only 27%. The common use of in-home treatment devices in this population is a likely explanation for this result [29] and the common use of these devices in North America [37-39] may, therefore, also complicate the use of this surrogate in other North American populations. Given such high misclassification, serious concerns may exist in studies that use home water source as surrogate for drinking water source, especially when exposure may be dependent on the use of water treatment devices.

This study demonstrated high misclassification errors associated with the use of home water source as a surrogate for drinking water source. Our findings corroborate those of others [1-4], which report that inaccuracy in waterborne exposure assessment is likely to occur from several sources including, but not limited to: individual variability in water consumption, bottled water use, use of treatment devices, and consumption of water outside of the home. Dodds et al. [18] also report that the U.S. Environmental Protection Agency has made recommendations to improve the exposure assessment in future epidemiologic studies on chlorination disinfection byproducts in drinking water, "by collecting individual level information on exposure to residential water...".

King et al. report that systematic reviews of the literature consistently identify non-differential exposure misclassification as a limiting factor in waterborne disinfection by-product risk estimation [19], which would result in a bias towards the null or an underestimate of a true effect. While this reduction in tendency for type-I errors is beneficial, the importance of conducting type-II errors should not be dismissed. For instance, Lynch et al. [1], state that " [misclassification error] is common in environmental epidemiology studies and its presence...indicates that a result of no association for an environmental exposure must be interpreted with caution, particularly if the potential exists for random misclassification of the exposure variable". Falsely concluding "no association" between a waterborne exposure and an adverse health outcome could prevent needed attention or policy changes, impair timely and appropriate public health response, and could contribute to the collection of conflicting results from waterborne disease investigations.

It is important to recognize that this discussion pertains only to the use of water for drinking purposes, and only water consumed plain or in re-constituted cold beverages; hence the use of this surrogate for non-consumption purposes (e.g. showering and bathing) may be appropriate. The misclassification errors might also be lower than those observed here, if the water system serving the individuals' alternate consumption locations (e.g. work, school etc.) was the same as that serving their home. Unfortunately, we were unable to evaluate this here. Overall however, the use of home water source as a surrogate measure of drinking water source was associated with high misclassification errors in this population.

## Conclusion

This study demonstrated substantial misclassification error associated with two surrogate measures of home water source and a commonly-used surrogate measure of individuals' drinking water sources. By applying our misclassification estimates to results from a previous study, we also illustrated the potential effect of exposure misclassification on the observed measures of association. While these surrogates are inexpensive and convenient, they are much less useful than direct collection of accurate data pertaining to the subjects' waterborne exposures, and significant misclassification may result from their use. Our results may serve as a reminder of the importance of collecting accurate, individual-level exposure data in studies of waterborne disease. They also lend support for further investigation into the quantification of waterborne exposure misclassification in other populations, which would provide useful estimates for consideration in interpretation of studies of waterborne disease. In studies where these surrogates must be used, estimation of the misclassification and its subsequent effects are recommended in the interpretation and communication of results.

## Competing interests

The author(s) declare that they have no competing interests.

## Authors' contributions

SEM designed and administered the survey, and collected the data. AQJ designed the study, performed the statistical analyses, and drafted the manuscript. CED, KD, SEM, SAMc and DWT provided critical feedback of the analyses and interpretation of results, as well as editorial comments on the manuscript.
